# 抗体偶联药物在晚期非小细胞肺癌中的研究进展

**DOI:** 10.3779/j.issn.1009-3419.2022.102.01

**Published:** 2022-03-20

**Authors:** 娜 王, 璐 赵, 豆 张, 英杰 贾, 凡铭 孔

**Affiliations:** 300193 天津，天津中医药大学第一附属医院肿瘤科，国家中医针灸临床医学研究中心 Department of Oncology, First Teaching Hospital of Tianjin University of Traditional Chinese Medicine; National Clinical Research Center for Chinese Medicine Acupuncture and Moxibustion, Tianjin 300193, China

**Keywords:** 肺肿瘤, 抗体偶联药物, 人表皮生长因子受体2, Lung neoplasms, Antibody-drug conjugates, Human epidermal growth factor receptor 2

## Abstract

肺癌是全球发病率和死亡率最高的恶性肿瘤之一，非小细胞肺癌（non-small cell lung cancer, NSCLC）是肺癌重要的病理类型之一，且晚期患者预后较差，内科治疗仍是其主要治疗手段。抗体偶联药物（antibody-drug conjugates, ADCs）是一类非常有潜力的新型抗肿瘤药物，由单克隆抗体和小分子细胞毒药物通过连接子偶联而成，在肺癌等实体瘤中应用前景广阔。本文对现阶段ADCs在晚期NSCLC中的作用机制和研究进展进行综述。

## Abstract (en)

Lung cancer is one of the malignant tumors with the highest morbidity and mortality in the world. Non-small cell lung cancer (NSCLC) is one of the most important pathological types of lung cancer. The prognosis of advanced NSCLC is poor and medical treatment is still the main treatment option. Antibody-drug conjugates (ADCs) are the kind of potentially new anti-tumor drugs, consisting of monoclonal antibodies conjugated to the cytotoxic payloads via the synthetic linkers. They have a broad application prospect in solid tumors such as lung cancer. This article focuses on the mechanism of action and research progress of ADCs in advanced NSCLC.

## 1 前言

肺癌是全球范围内对人类健康和生命威胁最大的恶性肿瘤之一^[1]^。我国肺癌的发病率和死亡率均位居榜首并有逐年攀升的趋势^[2]^，其中非小细胞肺癌（non-small cell lung cancer, NSCLC）约占肺癌的85%。约2/3的NSCLC患者在确诊时已为晚期，5年生存率不足15%^[3]^。除了传统的治疗方案如手术和放化疗之外，目前的治疗方案还包括靶向治疗、抗血管生成治疗和免疫检查点抑制剂等。尽管这些药物在晚期NSCLC的临床诊疗中展现出了较好的疗效和用药安全性，但都不可避免地出现了获得性耐药和原发性耐药等问题^[4-6]^。

近年来，抗体偶联药物（antibody-drug conjugates, ADCs）实现了小分子化疗和单抗药物靶向治疗以减毒增效为目的的强强联合，为实现肿瘤的“精准治疗”提供了一种崭新的途径^[7]^。ADCs药物与靶细胞特异性结合后形成ADC-抗原复合物，这种复合物通过网格蛋白介导被内吞作用内化进入靶细胞内部，从而形成一种含ADC-抗原复合物的初级内体；初级内体发展为次级内体后，ADCs药物与溶酶体结合，溶酶体中的质子泵会创造一种酸性环境，促进由蛋白酶（如cathepsin-B、plasmin）介导的蛋白水解裂解并允许细胞毒性载荷释放到靶细胞中；细胞毒性载荷通常通过诱导DNA损伤或干扰微管形成或分解从而引起靶细胞凋亡^[8-10]^。ADCs的抗体Fab段与癌细胞的抗原表位结合后，其Fc段与杀伤细胞[自然杀伤（natural killer, NK）细胞、巨噬细胞等]表面的Fc受体结合引发抗体依赖的细胞介导的细胞毒性作用（antibody-dependent cell-mediated cytotoxicity, ADCC），从而介导杀伤细胞直接杀伤癌细胞，并且抑制抗原受体下游信号传导将癌细胞阻滞在调定点，并通过释放促凋亡蛋白（如穿孔素和颗粒酶）诱导癌细胞死亡。

本文从ADCs在晚期NSCLC中的研究进程、临床应用及其所面临的问题进行综述，探讨ADCs的临床疗效、用药安全性及其应用前景（表 1）。

**表 1:** ADCs治疗晚期NSCLC的临床研究 Clinical trials of ADCs in the treatment of advanced NSCLC

ADCs	Clinical trial	Reference	Efficacy	Adverse events
T-DM1	UMIN000017709	Hotta K, 2018^[14]^	ORR: 6.7%; PFS: 2.0 mon; OS: 10.9 mon	Grade 3 or 4 thrombocytopenia (40%) and hepatotoxicity (20%), without any treatment-related deaths
DS-8201a	NCT03505710	Li BT, 2021^[22]^	ORR: 55%; DOR: 9.3 mon; PFS: 8.2 mon; OS: 17.8 mon	Grade 3 or higher neutropenia (19%) and adjudicated drug-related interstitial lung disease (26%) resulted in death in 2 patients
IMMU-132	NCT01631552	Heist RS, 2017^[26]^	ORR: 19%; DOR: 6.0 mon; CBR: 43%	Grade 3 or higher neutropenia (28%), diarrhea (7%), nausea (7%), fatigue (6%), and febrile neutropenia (4%)
ABBV-399	NCT03311477	Fujiwara Y, 2021^[32]^	ORR: 23%; DOR: 8.7 mon; PFS: 5.2 mon	Grade 3 or higher decreased neutrophil count and hypoalbuminemia (22% each)
PF-06647020	NCT02222922	Jasgit CS, 2018^[37]^	ORR: 16%; DCR: 56%; DOR: 5.8 mon; PFS: 2.9 mon	Grade 1 or 2 nausea, alopecia, fatigue, headache, neutropenia, and vomiting, without any treatment-related deaths
ADCs: antibody-drug conjugates; ORR: objective response rate; PFS: progression-free survival; OS: overall survival; DOR: duration of response; CBR: clinical benefit rate; DCR: disease control rate.				

## 2 ADCs在晚期NSCLC中的研究进展

### 2.1 靶向人表皮生长因子受体2（human epidermal growth factor receptor 2, HER2）的ADCs

HER2是表皮生长因子受体（epidermal growth factor receptor, EGFR）酪氨酸激酶家族的重要成员。本文重点讨论针对*HER2*阳性NSCLC的两个ADCs药物，即Trastuzumab emtansine（T-DM1）和Trastuzumab deruxtecan（DS-8201a）。

#### 2.1.1 T-DM1

T-DM1是第一个获得美国食品药品监督管理局（Food and Drug Administration, FDA）批准用于乳腺癌的ADCs药物，在晚期乳腺癌中展现了较为优越的有效性和安全性^[11]^。T-DM1在CALU-3肺癌细胞[HER2-免疫组化（immunohistonchemistry, IHC）3+]临床前研究中表现出剂量依赖性抑制肿瘤细胞生长作用^[12]^。此外，T-DM1在*HER2*突变外显子20插入突变的肺癌中治疗效果显著^[13]^。

一项关于T-DM1单药治疗*HER2*阳性NSCLC（IHC 3+，IHC 2+/荧光原位杂交阳性，或外显子20突变）的II期临床试验（UMIN000017709）^[14, 15]^由于疗效有限，提前终止。另一项关于T-DM1治疗*HER2*阳性晚期或转移性NSCLC的II期临床试验（NCT02289833）^[16]^也得出了相似的客观有效率（objective response rate, ORR）研究结果。根据IHC染色强度将纳入评估的49例患者分为两组（29例IHC 2+，20例IHC 3+），在IHC 2+队列中未观察到治疗反应，在IHC 3+队列中观察到4例患者部分缓解（ORR: 20%, 95%CI: 5.7%-43.7%）。两组患者的中位无进展生存期（progression-free survival, PFS）分别为2.6个月和2.7个月，中位总生存期（overall survival, OS）分别为12.2个月和12.1个月，组间比较均无差异。

综上，在NSCLC临床前研究中，T-DM1表现出较强的促肿瘤细胞凋亡和抗肿瘤细胞增殖作用，其作用水平取决于HER2的表达水平。在T-DM1治疗*HER2*阳性NSCLC的相关临床研究中，由于疗效有限，美国癌症协会、国家综合癌症网络（National Comprehensive Cancer Network Guidelines, NCCN）认为目前T-DM1可作为*HER2*突变的治疗方案，但尚未批准用于*HER2*阳性NSCLC的治疗^[3]^。

#### 2.1.2 DS-8201a

与T-DM1相比，DS-8201a具有更高的药物抗体比（drug-to-antibody ratio, DAR）和膜通透性，在内化进入靶细胞后能迅速水解并释放DXd^[17]^。体内外试验^[18]^结果表明DXd具有高度的膜通透性，DS-8201a可通过影响HER2低表达肿瘤细胞，而表现出旁观者效应。美国FDA先后批准DS-8201a用于后线治疗晚期*HER2*阳性乳腺癌、胃或胃食管交界处腺癌患者^[19, 20]^。

一项多中心、国际化的II期临床试验（DESTINY-Lung01, NCT03505710）中，共有91例*HER2*阳性NSCLC患者入组，ORR为55%，中位缓解持续时间（duration of response, DOR）为9.3个月（95%CI: 5.7-14.7），PFS为8.2个月（95%CI: 6.0-11.9），OS为17.8个月（95%CI: 13.8-22.1）。最常见的不良反应是中性粒细胞减少（19%），26%的患者发生药物相关的间质性肺病，并导致2例患者死亡^[21]^。

综上，DS-8201a在HER2阳性NSCLC中表现出较强的抗肿瘤活性和安全性。尽管在HER2阳性NSCLC患者中使用剂量为6.4 mg/kg的DS-8201a治疗展示了较为显著的抗肿瘤作用，DS-8201a在HER-2低表达患者中的治疗潜能以及安全性仍有待明确。

### 2.2 靶向人滋养层细胞表面糖蛋白抗原（trophoblast cell surface antigen 2, Trop-2）的ADCs：Sacituzumab govitecan（IMMU-132）

Trop-2介导的信号通路主要通过调节钙离子的信号通路、细胞周期蛋白表达及降低纤黏蛋白黏附作用以促进肿瘤细胞的增殖和转移^[22]^。IMMU-132是一种由靶向Trop-2抗原的人源化IgG1抗体通过可切割连接子偶联到伊立替康的活性代谢产物^[23]^，FDA授予其治疗转移性NSCLC和小细胞肺癌的快速通道认定^[24]^。

在一项关于IMMU-132治疗转移性NSCLC的单臂多中心研究试验（NCT01631552）中，54例转移性NSCLC受试者接受了在连续21 d为1个周期的第1天和第8天注射8 mg/kg或10 mg/kg剂量的IMMU-132的治疗。其ORR为19%，DOR为6.0个月（95%CI: 4.8-8.3），临床获益率（clinical benefit rate, CBR）为43%。9例意向治疗（intention-to-treat, ITT）受试者的ORR为17%，PFS为5.2个月（95%CI: 3.2-7.1）^[25]^。综上，IMMU-132具有良好的用药安全性和较为持久的反应持续时间，在针对NSCLC和其他Trop-2表达的肿瘤相关的治疗和预后状况仍有待进一步研究和探索。

### 2.3 靶向c-间充质上皮转化因子（mesenchymal epithelial transition, MET）的ADCs

MET是一种由*MET*原癌基因编码的受体酪氨酸激酶^[26, 27]^。*c-Met*基因的扩增或过度表达可能是肿瘤细胞对表皮生长因子受体（epidermal growth factor receptor, EGFR）酪氨酸激酶抑制剂（tyrosine kinase inhibitor, TKI）产生耐药的机制之一，通过激活ErbB3的EGFR非依赖性磷酸化和PI3K/AKT下游通路，在EGFR抑制剂存在的情况下提供一个旁路，从而导致EGFR-TKI耐药的发生^[28]^。

#### 2.3.1 Telisotuzumab vedotin（ABBV-399）

ABBV-399是一种由靶向c-Met的人源化单克隆抗体ABT-700通过缬氨酸-瓜氨酸连接子偶联微管蛋白抑制剂auristatin E（MMAE）组成的新型药物，其平均DAR为3.1^[29]^。ABT-700能以高亲和力特异性地将ABBV-399靶向c-Met表达的肿瘤细胞，通过抑制微管蛋白聚合而发挥其抑制肿瘤细胞有丝分裂和其他功能的作用，从而导致肿瘤细胞死亡^[30]^。

ABBV-399治疗晚期NSCLC的II期临床研究^[31]^（NCT03311477，受试者接受1.9 mg/kg每2周1次或2.7 mg/kg每3周注射1次）表明：52例受试者中有40例进入疗效评估人群。其中ORR为23%，DOR为8.7个月，PFS为5.2个月。目前，一项关于ABBV-399在*c-Met*阳性NSCLC患者后线治疗的II期临床试验（NCT03539536）正在进行中，有望为ABBV-399的疗效提供进一步的证据。

#### 2.3.2 SHR-A1403

SHR-A1403是一种由靶向c-Met的人源化IgG2单克隆抗体偶联新型细胞毒性微管抑制剂而组成的药物，其DAR为2^[32]^。一项临床前研究^[33]^表明SHR-A1403在c-Met过表达的细胞中有效地克服了AZD9291的耐药性，另一项研究^[34]^首次报道了SHR-A1403在胰腺导管腺癌临床前模型中的应用前景，SHR-A1403显著抑制胰腺癌细胞的增殖、迁移和侵袭，诱导细胞周期阻滞和凋亡。目前，一项关于SHR-A1403在晚期实体肿瘤患者中安全性和耐受性的I期临床试验（NCT03856541）正在进行中，这些患者包括对标准治疗无效的NSCLC患者。

### 2.4 靶向蛋白酪氨酸激酶7（protein tyrosine kinase 7, PTK7）的ADCs：PF-06647020（Cofetuzumab pelidotin）

PTK7是一种缺乏催化活性的受体蛋白酪氨酸激酶（receptor protein tyrosine kinase, RTK）^[35]^。PF-06647020是一种由靶向PTK7的人源化IgG1单克隆抗体即hu6M024，通过可切割的缬氨酸-瓜氨酸连接子偶联微管抑制剂auristatin-0101（Au0101）而组成的新型药物，其DAR为4^[36]^。

在一项评估PF-06647020对标准治疗耐药的晚期实体肿瘤患者治疗相关的安全性和有效性的I期临床试验（NCT02222922）^[37]^中，符合条件的受试者按晚期卵巢癌、NSCLC和三阴性乳腺癌在内的剂量扩增队列接受PF-06647020静脉注射治疗，每3周1次。试验结果表明：在II期临床试验中推荐剂量为2.8 mg/kg；在25例NSCLC中，ORR为16%，DCR为56%，DOR为5.8个月，PFS为2.9个月。值得注意的是，肿瘤组织中PTK7的表达水平处于中高水平，提示PTK7的表达与PF-06647020的临床疗效之间可能存在线性相关。此外，其他ADCs诸如CX-2009、XMT-1536、Enapotamab vedotin等也在临床研究中，初步研究结果显示了较好的疗效和应用前景。

## 3 结语

ADCs在晚期NSCLC的临床研究中展现出较好的治疗效果和安全性，为晚期NSCLC的个性化和精准治疗提供了一种方案选择。但仍存在诸如半衰期短、DAR不均质、偶联位点杂乱等不利的药代动力学特征和非靶向效应。相信随着对ADCs研究不断深入和各学科技术不断发展，会有更多的患者从中获益。

## References

[1] Siegel RL, Miller KD, Fuchs HE (2021). Cancer statistics, 2021. CA Cancer J Clin.

[2] Cao M, Chen W (2019). Epidemiology of lung cancer in China. Thorac Cancer.

[3] Ettinger DS, Wood DE, Aisner DL (2021). NCCN guidelines insights: non-small cell lung cancer, version 2. 2021. J Natl Compr Canc Netw.

[4] Meador CB, Hata AN (2020). Acquired resistance to targeted therapies in NSCLC: Updates and evolving insights. Pharmacol Ther.

[5] Rotow J, Bivona TG (2017). Understanding and targeting resistance mechanisms in NSCLC. Nat Rev Cancer.

[6] Wu SG, Shih JY (2018). Management of acquired resistance to EGFR TKI-targeted therapy in advanced non-small cell lung cancer. Mol Cancer.

[7] Sievers EL, Senter PD (2013). Antibody-drug conjugates in cancer therapy. Annu Rev Med.

[8] Zolot RS, Basu S, Million RP (2013). Antibody-drug conjugates. Nat Rev Drug Discov.

[9] Lambert JM, Berkenblit A (2018). Antibody-drug conjugates for cancer treatment. Annu Rev Med.

[10] Birrer MJ, Moore KN, Betella I (2019). Antibody-drug conjugate-based therapeutics: state of the science. J Natl Cancer Inst.

[11] Nakada T, Masuda T, Naito H (2016). Novel antibody drug conjugates containing exatecan derivative-based cytotoxic payloads. Bioorg Med Chem Lett.

[12] Lewis Phillips GD, Li G, Dugger DL (2008). Targeting *HER2*-positive breast cancer with trastuzumab-DM1, an antibody-cytotoxic drug conjugate. Cancer Res.

[13] Perera SA, Li D, Shimamura T (2009). HER2YVMA drives rapid development of adenosquamous lung tumors in mice that are sensitive to BIBW2992 and rapamycin combination therapy. Proc Natl Acad Sci U S A.

[14] Hotta K, Aoe K, Kozuki T (2018). A phase II study of trastuzumab emtansine in *HER2*-positive non-small cell lung cancer. J Thorac Oncol.

[15] Verma S, Miles D, Gianni L (2012). Trastuzumab emtansine for *HER2*-positive advanced breast cancer. N Engl J Med.

[16] Peters S, Stahel R, Bubendorf L (2019). Trastuzumab emtansine (T-DM1) in patients with previously treated HER2-overexpressing metastatic non-small cell lung cancer: Efficacy, safety, and biomarkers. Clin Cancer Res.

[17] Ogitani Y, Aida T, Hagihara K (2016). DS-8201a, a novel HER2-targeting ADC with a novel DNA Topoisomerase I inhibitor, demonstrates a promising antitumor efficacy with differentiation from T-DM1. Clin Cancer Res.

[18] Doi T, Shitara K, Naito Y (2017). Safety, pharmacokinetics, and antitumour activity of trastuzumab deruxtecan (DS-8201), a HER2-targeting antibody-drug conjugate, in patients with advanced breast and gastric or gastro-oesophageal tumours: a phase 1 dose-escalation study. Lancet Oncol.

[19] Zhu Y, Zhu X, Wei X (2021). HER2-targeted therapies in gastric cancer. Biochim Biophys Acta Rev Cancer.

[20] Narayan P, Osgood CL, Singh H (2021). FDA approval summary: Fam-Trastuzumab Deruxtecan-Nxki for the treatment of unresectable or metastatic *HER2*-positive breast cancer. Clin Cancer Res.

[21] Li BT, Smit EF, Goto Y (2022). Trastuzumab deruxtecan in *HER2*-mutant non-small-cell lung cancer. N Engl J Med.

[22] Goldenberg DM, Stein R, Sharkey RM (2018). The emergence of trophoblast cell-surface antigen 2 (TROP-2) as a novel cancer target. Oncotarget.

[23] Starodub AN, Ocean AJ, Shah MA (2015). First-in-human trial of a novel anti-Trop-2 antibody-SN-38 conjugate, Sacituzumab Govitecan, for the treatment of diverse metastatic solid tumors. Clin Cancer Res.

[24] Syed YY (2020). Sacituzumab govitecan: first approval. Drugs.

[25] Heist RS, Guarino MJ, Masters G (2017). Therapy of advanced non-small-cell lung cancer with an SN-38-anti-trop-2 drug conjugate, sacituzumab govitecan. J Clin Oncol.

[26] Awad MM, Oxnard GR, Jackman DM (2016). *MET* exon 14 mutations in non-small-cell lung cancer are associated with advanced age and stage-dependent *MET* genomic amplification and c-Met overexpression. J Clin Oncol.

[27] Huang C, Zou Q, Liu H (2020). Management of non-small cell lung cancer patients with *MET* exon 14 skipping mutations. Curr Treat Options Oncol.

[28] Wang Q, Yang S, Wang K (2019). MET inhibitors for targeted therapy of EGFR TKI-resistant lung cancer. J Hematol Oncol.

[29] Strickler JH, Weekes CD, Nemunaitis J (2018). First-in-human phase I, dose-escalation and -expansion study of Telisotuzumab vedotin, an antibody-drug conjugate targeting c-Met, in patients with advanced solid tumors. J Clin Oncol.

[30] Wang J, Anderson MG, Oleksijew A (2017). ABBV-399, a c-Met antibody-drug conjugate that targets both *MET*-amplified and c-Met-overexpressing tumors, irrespective of MET pathway dependence. Clin Cancer Res.

[31] Fujiwara Y, Kenmotsu H, Yamamoto N (2021). Phase 1 study of telisotuzumab vedotin in Japanese patients with advanced solid tumors. Cancer Med.

[32] Yang C, Zhao X, Sun X (2019). Preclinical pharmacokinetics of a novel anti-c-Met antibody-drug conjugate, SHR-A1403, in rodents and non-human primates. Xenobiotica.

[33] Tong M, Gao M, Xu Y (2019). SHR-A1403, a novel c-mesenchymal-epithelial transition factor (c-Met) antibody-drug conjugate, overcomes AZD9291 resistance in non-small cell lung cancer cells overexpressing c-Met. Cancer Sci.

[34] Yang CY, Wang L, Sun X (2019). SHR-A1403, a novel c-Met antibody-drug conjugate, exerts encouraging anti-tumor activity in c-Met-overexpressing models. Acta Pharmacol Sin.

[35] Chen R, Khatri P, Mazur PK (2014). A *meta*-analysis of lung cancer gene expression identifies PTK7 as a survival gene in lung adenocarcinoma. Cancer Res.

[36] Maitland ML, Sachdev JC, Sharma MR (2021). First-in-human study of PF-06647020 (Cofetuzumab Pelidotin), an antibody-drug conjugate targeting protein tyrosine kinase 7, in advanced solid tumors. Clin Cancer Res.

[37] Jasgit CS, Michael LM, Manish S (2018). PF-06647020 (PF-7020), an antibody-drug conjugate (ADC) targeting protein tyrosine kinase 7 (PTK7), in patients (pts) with advanced solid tumors: Results of a phase I dose escalation and expansion study. J Clin Oncol.

